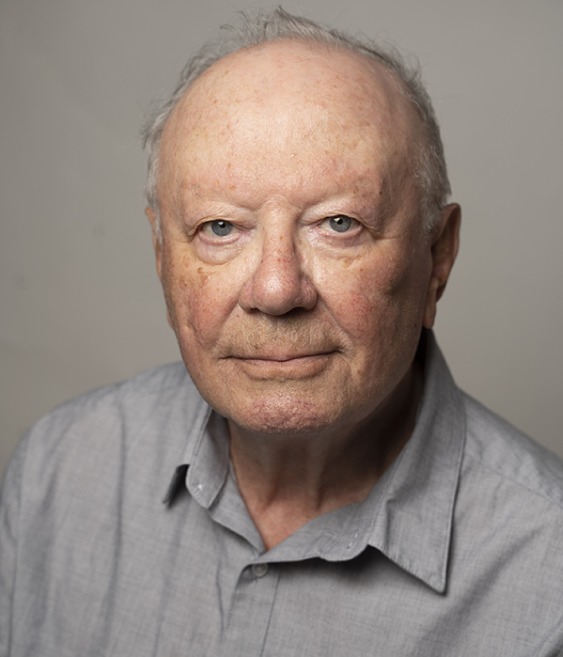# Preface to ‘Modelling of dynamic phenomena and localization in structured media (part 2)’

**DOI:** 10.1098/rsta.2019.0471

**Published:** 2019-11-25

**Authors:** G. Mishuris, A. Movchan, L. Truskinovsky

This two-volume special issue of *Philosophical Transactions of the Royal Society A* presents a broad panorama of topics in mechanics of solids and wave mechanics influenced and largely shaped by the seminal contributions of Leonid Slepyan, whose 90th birthday we have recently celebrated.

The first volume, containing our comments on the life and career of Leonid Slepyan, addresses important questions in fracture mechanics, dynamics of elastic composites, mechanics of vibrations, dispersive systems and dynamic localization.

The second volume, which we introduce here, addresses various topics related to wave propagation in discrete systems, nonlinear dynamic phenomena, mechanics of internal activity, chirality systems, matrix Wiener–Hopf methods and mechanics of quasi-crystallinity.

It has been our privilege to work with Leonid Slepyan, and we would like to take this opportunity to wish him many more years of inspiring and productive work.

**Figure:**